# Exercise-Induced Oxygen Desaturation and Outcomes After Nintedanib Therapy for Fibrosing Interstitial Lung Disease in Patients Without Dyspnea

**DOI:** 10.3390/jcm13247865

**Published:** 2024-12-23

**Authors:** Masaki Okamoto, Toyoshi Yanagihara, Kiminori Fujimoto, Tomonori Chikasue, Kazuhiro Tabata, Yoshiaki Zaizen, Masaki Tominaga, Akiko Sumi, Yuuya Nishii, Norikazu Matsuo, Takashi Nouno, Shuji Matsuura, Atsushi Kawaguchi, Tomoaki Hoshino

**Affiliations:** 1 Department of Respirology, NHO Kyushu Medical Center, Jigyohama 1-8-1, Chuo-ku, Fukuoka 810-0065, Japan; 2 Division of Respirology, Neurology and Rheumatology, Department of Internal Medicine, Kurume University School of Medicine, Asahi-Machi 67, Kurume 830-0011, Japan; 3 Department of Respiratory Medicine, Fukuoka University Hospital, 7-45-1 Nanakuma, Jonan-ku, Fukuoka 814-0180, Japan; 4 Department of Radiology, Kurume University School of Medicine, Asahi-Machi 67, Kurume 830-0011, Japan; 5 Department of Pathology, Kagoshima University Graduate School of Medical and Dental Sciences, 8-35-1 Sakuragaoka, Kagoshima 890-8544, Japan; 6 Department of Radiology, NHO Kyushu Medical Center, Jigyohama 1-8-1, Chuo-ku, Fukuoka 810-0065, Japan; 7 Education and Research Center for Community Medicine, Faculty of Medicine, Saga Medical School, 5-1-1 Nabeshima, Saga 849-8501, Japan

**Keywords:** fibrosing interstitial lung disease, nintedanib, dyspnea

## Abstract

**Background:** The degree of exercise-induced oxygen desaturation and outcomes following antifibrotic drug therapy in asymptomatic patients with fibrosing interstitial lung disease (FILD) remain unclear. **Methods:** We compared clinical data, incidence of annual FILD progression, overall survival, and tolerability after initiating nintedanib between 58 patients with dyspnea and 18 patients without. Annual FILD progression was defined as >10% decrease in forced vital capacity (FVC), >15% decrease in diffusing capacity of the lungs for carbon monoxide (D_LCO_), developing acute exacerbations, or FILD-related death within 1 year of starting nintedanib. Outcomes between the two groups were adjusted for covariates, including age, gender, FVC, D_LCO_, and diagnosis of idiopathic pulmonary fibrosis, all known prognostic factors for FILD. **Results:** In 6-min walk test, incidence of decrease to <90% of SpO_2_ was significantly lower in non-dyspnea group than in dyspnea group (24% vs. 55%, *p* = 0.028), but incidence of >4% decreases showed no significant difference (71% vs. 89%, *p* = 0.11) The incidence of annual progression was significantly lower in non-dyspnea than in dyspnea group (17% vs. 53%, adjusted *p* = 0.026). The relative change in D_LCO_ was significantly slower in non-dyspnea group (adjusted *p* = 0.036), but FVC was not (adjusted *p* = 0.067). Overall survival was longer in non-dyspnea group (adjusted *p* = 0.0089). The discontinuation rate and therapeutic period of nintedanib were not significantly different between the groups. **Conclusions:** Asymptomatic patients with FILD have severe exercise-induced oxygen desaturation and better outcomes after nintedanib therapy than symptomatic patients. Antifibrotic drug therapy should not be avoided solely because of a lack of symptoms.

## 1. Introduction

Fibrosing interstitial lung disease (FILD) encompasses various subtypes characterized by specific patterns of lung parenchymal fibrosis with or without inflammation [1,2,3,4]. Idiopathic pulmonary fibrosis (IPF), which pathologically manifests as usual interstitial pneumonia (UIP), is the most common type of idiopathic interstitial pneumonia [1,2,3,4]. Patients with IPF have a grave prognosis with a median survival of approximately 3 years [5]. A proportion of patients with non-IPF FILD exhibit progressive characteristics, defined as progressive pulmonary fibrosis by global guidelines and various studies, and have similarly poor survival rates as patients with IPF [1,6]. Two randomized controlled trials and one meta-analysis have shown that the antifibrotic agents nintedanib and pirfenidone slow the decline in forced vital capacity (FVC), reduce the incidence of acute exacerbation (AE) and/or lower all-cause mortality in patients with IPF [7,8,9]. The recent phase 3 INBUILD trial revealed that nintedanib reduced the decline in FVC in patients with progressive non-IPF FILD [10]. Consequently, both the global guidelines and the Japanese guidelines moderately recommend antifibrotic drug therapy for these patients [1,11].

Recently, some studies have suggested the effectiveness of antifibrotic drugs in patients with early FILD [12,13]. In the INMARK, a phase 3 trial involving patients with early IPF who had an FVC of >80%, nintedanib reduced the decrease in FVC for 12 weeks [12]. In the subgroup analysis of the INBUILD trial, nintedanib suppressed the decrease in FVC over 52 weeks regardless of severity markers, such as the gender–age–pulmonary physiology (GAP) score and baseline FVC [10,13]. However, antifibrotic drug therapy for patients without dyspnea is often avoided because patients and respiratory physicians often consider such therapy to be unnecessary before the disease becomes severe. In fact, a lower score on the modified Medical Research Council (mMRC) scale evaluating the severity of dyspnea in daily life was associated with better survival in patients with IPF [14]. By contrast, in the subgroup analysis of the SENSCIS, a phase 3 trial involving patients with systemic sclerosis (SSc)-associated ILD, the nintedanib group showed a numerically greater reduction in the FVC decline compared with placebo in patients without than with dyspnea, although the difference was not statistically significant [15]. The high effectiveness of antifibrotic drugs in asymptomatic patients with FILD may be associated with the presence of potential exercise-induced oxygen desaturation. Desaturation during the 6 min walk test (6MWT) has been reported as a predictor of survival and/or disease progression in patients with IPF [16,17], especially in those with early-stage disease classified as stage I in the Japanese Respiratory Society disease severity system [18,19]. Nishiyama et al. [20] suggested that dyspnea may not present in patients with FILD despite high disease severity, because patients with IPF may experience more severe desaturation but milder dyspnea than those with chronic obstructive pulmonary disease (COPD).

In the present study, we attempted to clarify the degree of exercise-induced oxygen desaturation and the outcomes following nintedanib therapy in patients with FILD without dyspnea.

## 2. Materials and Methods

### 2.1. Patient Selection

We retrospectively analyzed consecutive patients with FILD who received nintedanib therapy in our hospital from 2015 to 2022. The inclusion criteria were as follows: patients diagnosed with FILD according to a multidisciplinary discussion based on global guidelines on IPF, idiopathic interstitial pneumonia, SSc, polymyositis, rheumatoid arthritis, and Sjögren’s syndrome [1,4,21,22,23,24]. The exclusion criteria were patients who could not regularly visit our hospital at least 1 year after starting nintedanib and patients who could not undergo pulmonary function testing at least every 6 months, because the annual progression of FILD could not be accurately analyzed in such patients. AE of FILD was diagnosed in accordance with the criteria detailed in a previous report [25]. Disease severity was staged in accordance with the GAP model, classifying patients into three stages based on clinical variables, e.g., sex or age, FVC, and the diffusing capacity of the lung for carbon monoxide (D_LCO_)] [26]. The GAP score and chronic pulmonary emphysema assessment test (CAT) result, as patient-reported outcomes [27,28], were collected upon starting nintedanib. According to a previous report, the patients were classified into four groups based on their GAP scores: 0 or 1, 2 or 3, 4 or 5, and 6 to 8, with increasing severity [26].

### 2.2. Study Protocol

The patients’ clinical data upon starting nintedanib were collected to determine factors predicting the progression of FILD. The mMRC scale includes five grades (0–4) and is used to evaluate dyspnea in daily life [14]. Patients with an mMRC scale score of 0 were classified into the non-dyspnea group, and the remaining patients were classified into the dyspnea group. In the present study, a single physical therapist used the mMRC scale to evaluate the patients.

Annual progression of FILD was defined as a >10% decrease in FVC or a >15% decrease in D_LCO_, the development of AE, or death of FILD at 1 year after starting nintedanib. Overall survival (OS) was measured from the day of starting nintedanib to the final observation day (24 February 2024). When measuring OS, events were defined in two ways: death from any cause and FILD.

The treatment protocol with nintedanib was based on recommendations provided in the INPULSIS trials [7]. The recommended starting dose was 150 mg twice a day. Dose reductions of up to 100 mg twice a day and treatment interruptions were recommended to manage adverse events. After the improvement of adverse events, nintedanib could be reintroduced at the discretion of the physician.

### 2.3. Pulmonary Function Tests

Pulmonary function tests and measurement of FVC and forced expiratory volume in 1 s were performed using a spirometer (CHESTAC-8900, Chest, Tokyo, Japan). The D_LCO_ was measured using the single-breath technique. These techniques were performed according to the guidelines of the American Thoracic Society/European Respiratory Society [29].

### 2.4. 6MWT

The 6MWT was performed according to the American Thoracic Society statement [30]. The patients were instructed to walk as fast as possible and were allowed to slow down or stop if necessary during examination. The SpO_2_ and 6MWT distance were monitored and recorded.

### 2.5. Statistical Analysis

The data are expressed as the median (25th–75th percentiles of the interquartile range) or least squares (LS) mean ± standard error (SE) as appropriate. We analyzed the differences in clinical data between the non-dyspnea and dyspnea groups using the Wilcoxon rank-sum test, Fisher’s exact test, and multiple regression analysis as appropriate. The cut-off levels of the lowest and decreased SpO_2_ and the CAT score for comparison between the two groups were set at 90%, 4%, and 10, respectively. These levels were based on previous reports, indicating that they were prognostic factors of FILD or COPD [17,18,28]. In the logistic regression analysis using backward elimination and multiple regression analysis, we adjusted for covariates including age, gender, FVC, D_LCO_, and diagnosis with IPF as prognostic factors based on previous reports [1,2,3,26]. In the Kaplan–Meier analysis of OS using the log-rank test, we adjusted for the above covariates by Cox hazard proportional analysis with or without backward elimination. In the logistic regression analysis, a *p* value of <0.05 in the univariate analysis was set as the criterion for variable selection in the multivariate analysis. A *p* value of <0.05 was considered statistically significant. All statistical analyses were performed using JMP 14.0 (SAS Institute Japan, Tokyo, Japan).

## 3. Results

### 3.1. Patient Selection and Characteristics

**Figure 1:**
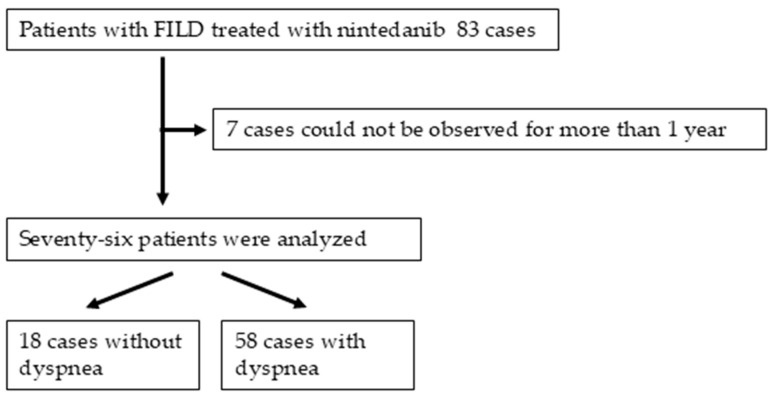
Inclusion and exclusion criteria for study patients. FILD, fibrosing interstitial pneumonia.

**Figure 2:**
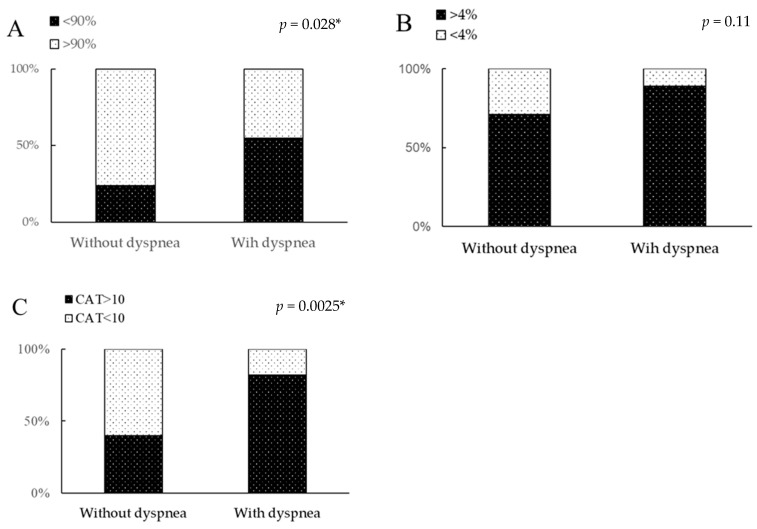
Comparison of desaturation and patient-reported outcomes among patients with FILD with versus without dyspnea. Comparison of (**A**) lowest and (**B**) highest decrease in SpO_2_ in the 6MWT and (**C**) CAT score between non-dyspnea and dyspnea groups. Cut-off levels were set at 90% of the lowest SpO_2_ in the 6MWT, 4% for the decrease in SpO_2_ in the 6MWT, and 10 for the CAT score as prognostic factors of FILD or chronic obstructive pulmonary disease based on previous reports. 6MWT, 6-min walk test; CAT, chronic obstructive pulmonary disease assessment test. * *p* < 0.05 is regarded as statistically significant.

The patient selection process based on the inclusion and exclusion criteria is shown in Figure 1. Among 83 identified patients with FILD, 7 patients did not meet the study criteria because of failure to undergo observation for more than 1 year. In total, 76 patients were selected for the study, including 56 (74%) male patients and 60 (79%) smokers, with a median age of 72.2 (65.9–76.2) years. Eighteen (24%) of seventy-three patients were assigned to the non-dyspnea group. The patients’ characteristics and comparison of clinical data upon starting nintedanib between the non-dyspnea and dyspnea groups are shown in Table 1 and Figure 2. The patients’ FILD subtypes included IPF in 53 (70%) patients, unclassifiable ILD in 11 (15%), fibrotic hypersensitivity pneumonia in 7 (9%), connective tissue disease-associated ILD (CTD-ILD) in 4 (5%), and smoking-related ILD in 1 (1%). Among the four patients with CTD-ILD, the disease subtypes of CTD were SSc, polymyositis, rheumatoid arthritis, and SSc with secondary Sjögren’s syndrome, in one patient each. According to the GAP models, the patients’ disease severity was classified as 0 or 1 in 9 (12%) patients, 2 or 3 in 38 (50%), 4 or 5 in 28 (37%), and 6 to 8 in 1 (1%). The radiological ILD patterns based on the global IPF guidelines [1] were definite UIP in 25 (33%) patients, probable UIP in 17 (22%), indeterminate for UIP in 21 (28%), and an alternative diagnosis in 13 (17%). Among the 73 patients, none had pulmonary hypertension. In the comparison of clinical data between the two groups, the non-dyspnea group included significantly more male patients; more patients with a lower mMRC scale score (median, 0 vs. 1), GAP score (median, 3 vs. 3), decrease in SpO_2_ (median, 5.0% vs. 7.0%) during the 6MWT, C-reactive protein level (median, 0.11 vs. 0.25 mg/dL), Krebs von den Lungen-6 (KL-6) level (median, 584.5 vs. 875.0 IU/mL), monocyte count, and CAT score (median, 6.0 vs. 17.0); and more patients with a higher FVC (median, 84.2% vs. 78.8%), D_LCO_ (median, 72.2% vs. 58.2%) and the lowest SpO_2_ (median, 92.0% vs. 88.0%) during the 6MWT compared to the dyspnea group (*p* < 0.05). Among the 76 patients, the incidence of a decrease of up to <90% in the SpO_2_ during the 6MWT was significantly lower in the non-dyspnea group than in the dyspnea group, but not significantly (24% vs. 55%, *p* = 0.028) (Table 1 and Figure 2A). The incidence of a >4% decrease in SpO_2_ during the 6MWT was not significantly different between the non-dyspnea and dyspnea groups (71% vs. 89%, *p* = 0.11) (Table 1 and Figure 2B). The rate of patients with a CAT score of >10 was significantly lower in the non-dyspnea group than in the dyspnea group (40% vs. 82%, *p* = 0.0025) (Table 1 and Figure 2C). There was no difference in the percentage of patients with a reduced starting dose of nintedanib between the non-dyspnea and dyspnea groups (6% vs. 10%, *p* = 1.0). The present study suggests that a certain proportion of patients who have FILD without dyspnea and require nintedanib therapy have severe exercise-induced oxygen desaturation.

### 3.2. Patient Outcomes

**Figure 3:**
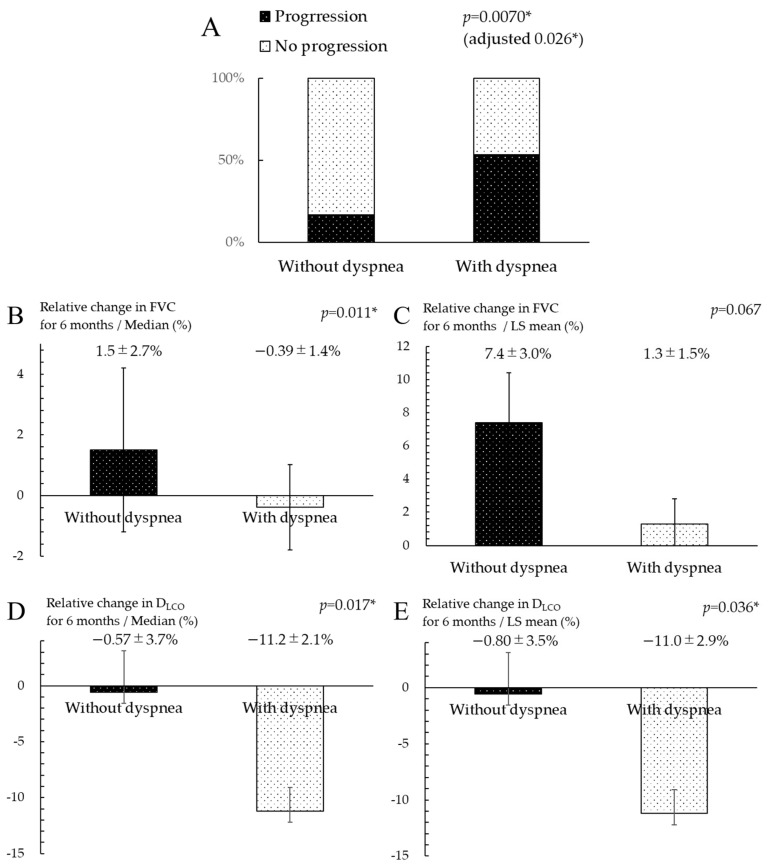

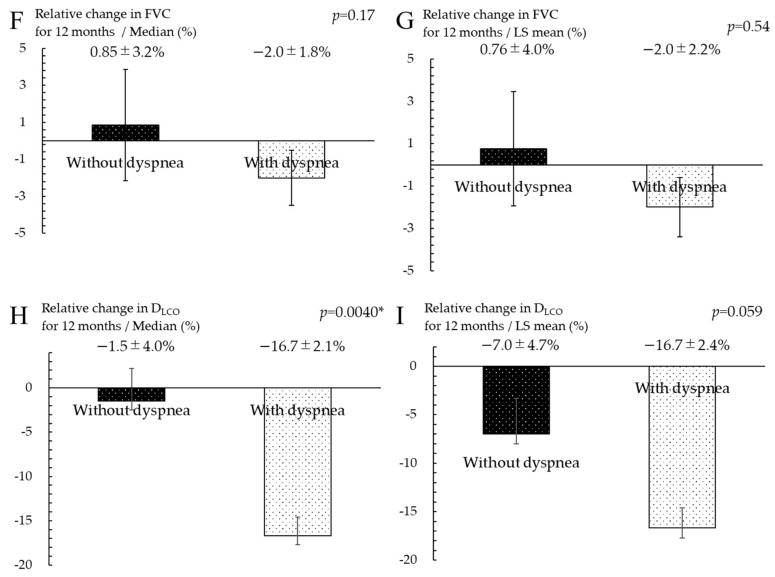
Comparison of progression among patients with FILD with versus without dyspnea. (**A**) Incidence of annual progression (defined as >10% relative decrease in FVC or 15% decrease in D_LCO_, development of acute exacerbation, or death) was compared among patients with FILD with versus without dyspnea using Fisher’s exact test. Adjustment for covariates including age, gender, FVC, D_LCO_, and diagnosis with IPF as prognostic factors based on previous reports was performed using logistic regression analysis. We compared the relative decrease in (**B**,**C**) FVC and (**D**,**E**) D_LCO_ over 6 months using the (**B**,**D**) Wilcoxon rank-sum test or (**C**,**E**) multiple regression analysis. Similarly, we compared the relative decrease in (**F**,**G**) FVC and (**H**,**I**) D_LCO_ over 12 months using the (**F**,**H**) Wilcoxon rank-sum test or (**G**,**I**) multiple regression analysis. Decreases in FVC and D_LCO_ are shown as (**B**,**D**,**F**,**H**) median ± SE or (**C**,**E**,**G**,**I**) LS mean ± SE. Adjustment for covariates including age, sex, FVC, D_LCO_, and diagnosis with IPF was performed using multiple regression analysis. FILD, fibrosing interstitial lung disease; FVC, forced vital capacity; D_LCO_, diffusing capacity of the lung for carbon monoxide; IPF, idiopathic pulmonary fibrosis; SE, standard error; LS, least squares. * *p* < 0.05 is regarded as statistically significant.

A comparison of the patients’ outcomes between the non-dyspnea and dyspnea groups is shown in Table 2. The median observation period was 861.0 (671.5–1488.5) days after starting nintedanib. Among the 76 patients, 24 (32%) and 17 (22%) patients died from any cause and from FILD, respectively. Compared with the dyspnea group, the non-dyspnea group showed significantly longer OS and significantly lower mortality from any cause and FILD (*p* < 0.05). In total, 34 (45%) of 76 patients exhibited annual progression (Table 2). Among the 76 patients, annual progression of FILD occurred in 3 (17%) of 18 patients in the non-dyspnea group and 31 (53%) of 58 patients in the dyspnea group, with a significant difference according to Fisher’s exact test (*p* = 0.0070). In a similar comparison of the components of annual progression of FILD, only the incidence of a >15% decrease in D_LCO_ was significantly lower in the non-dyspnea group than in the dyspnea group (*p* = 0.026); the other components showed no significant differences between the two groups. Moreover, the incidence of annual progression of FILD was also significantly lower in the non-dyspnea group than in the dyspnea group after adjusting for covariates (adjusted *p* = 0.026) (Figure 3A). Comparisons of the relative decrease in FVC and D_LCO_ over 6 months are shown in Figure 3B–E. The relative decrease in FVC (median ± SE, 1.5% ± 2.7% vs. −0.39% ± 1.4%; *p* = 0.011) and D_LCO_ (median ± SE, −0.57% ± 3.7% vs. −11.2% ± 2.1%; *p* = 0.017) were significantly slower in the non-dyspnea group than in the dyspnea group. After adjusting for covariates, the relative change in D_LCO_ was also significantly slower in the non-dyspnea group than in the dyspnea group (LS mean ± SE, −0.80% ± 3.5% vs. −11.0% ± 2.9%; *p* = 0.036). Similarly, the relative change in FVC tended to be slower in the non-dyspnea group than in the dyspnea group (LS mean ± SE, 7.4% ± 3.0% vs. 1.3% ± 1.5%; *p* = 0.067), but not significantly. Comparisons of the relative decrease in pulmonary function over 12 months are shown in Figure 3F–I. The relative decrease in D_LCO_ was significantly slower in the non-dyspnea group than in the dyspnea group (median ± SE, −1.5% ± 4.0% vs. −16.7% ± 2.1%; *p* = 0.0040), but the relative change in FVC showed no difference between the two groups (median ± SE, 0.85% ± 3.2% vs. −2.0% ± 1.8%; *p* = 0.17). After adjusting for covariates, the relative change in D_LCO_ tended to be slower in the non-dyspnea group than in the dyspnea group (LS mean ± SE, −7.0% ± 4.7% vs. −16.7% ± 2.4%; *p* = 0.059), but not significantly. Similarly, there was no significant difference in the relative change in FVC between the non-dyspnea and dyspnea group (LS mean ± SE, 0.76% ± 4.0% vs. −2.0% ± 2.2%; *p* = 0.54).

**Figure 4:**
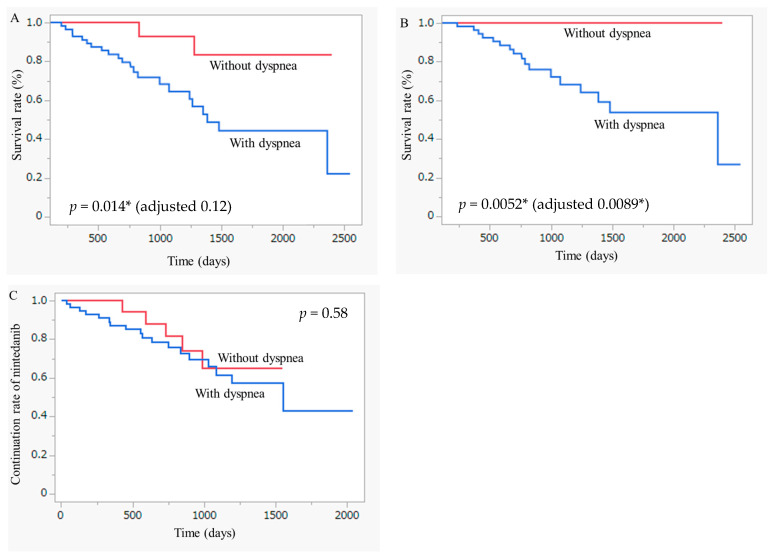
Comparison of survival between patients with FILD with versus without dyspnea. Kaplan–Meier curves for mortality from (**A**) any cause and (**B**) FILD after start of nintedanib between patients who had FILD with versus without dyspnea using the log-rank test. Adjustment for covariates including age, sex, FVC, D_LCO_, and diagnosis with IPF was performed using a Cox proportional hazard model. (**C**) Kaplan–Meier curves comparing the therapeutic period of nintedanib between patients with FILD with versus without dyspnea using the log-rank test. FILD, fibrosing interstitial lung disease; FVC, forced vital capacity; D_LCO_, diffusing capacity of the lung for carbon monoxide; IPF, idiopathic pulmonary fibrosis. * *p* < 0.05 is regarded as statistically significant.

The Kaplan–Meier curve is shown in Figure 4A,B. OS is defined by death from any cause (Figure 4A), and FILD (Figure 4B) was significantly longer in the non-dyspnea group than in the dyspnea group according to the log-rank test (*p* = 0.014 and *p* = 0.0052). After adjustment for covariates, the non-dyspnea group showed significantly longer OS defined by death due to FILD (adjusted *p* = 0.0089) than the dyspnea group. However, no significant difference in OS defined by death from any cause was observed between the two groups (adjusted *p* = 0.11).

In summary, patients with FILD without dyspnea had a significantly lower incidence of annual progression and mortality and a slower short-term decrease in pulmonary function after starting nintedanib than patients with dyspnea, even after adjustment for covariates.

### 3.3. Analysis of Risk Factors for Annual Progression of FILD

The results of the analysis of risk factors for the annual progression of FILD are shown in Table 3. The univariate analysis showed that the absence of dyspnea (odds ratio (OR), 0.17; 95% confidence interval (CI), 0.037–0.60; *p* = 0.011), higher lowest SpO_2_ during the 6MWT (OR, 0.90; 95% CI, 0.82–0.98; *p* = 0.021), lower decrease in SpO_2_ (OR, 1.1; 95% CI, 1.0–1.3; *p* = 0.029), and longer 6MWT distance (OR, 0.95; 95% CI, 0.90–0.99; *p* = 0.032) were significantly associated with a lower incidence of annual progression of FILD (Table 3A). We next assessed the results of the multivariate analysis with or without backward elimination including variables with a *p*-value of <0.05 in the univariate analysis and covariates. In the multivariate analysis with backward elimination, only the absence of dyspnea was independently associated with a lower incidence of annual progression (OR, 0.17; 95% CI, 0.045–0.67; *p* = 0.011) (Table 3B). In the multivariate analysis without backward elimination, the absence of dyspnea (OR, 0.14; 95% CI, 0.022–0.83; *p* = 0.031) and no diagnosis of IPF (OR, 4.4; 95% CI, 1.1–16.6; *p* = 0.031) were independently associated with a lower incidence of annual progression (Table 3C).

### 3.4. Tolerability of Nintedanib Therapy in Dyspnea and Non-Dyspnea Groups

The results of the analysis of tolerability of nintedanib are shown in Table 4. The comparison of the nintedanib therapeutic period between the two groups is shown in Figure 4C.

Among the 76 patients, the incidences of discontinuing nintedanib for all causes and because of side effects were 26 (34%) and 20 (26%) patients throughout the overall period. Similarly, annual discontinuation was experienced in 10 (13%) patients after starting nintedanib and was due to side effects in all of these patients. Interruption and dose reduction in nintedanib were experienced in 27 (36%) and 52 (68%) patients throughout the overall period. Causes of discontinuing nintedanib were worsening FILD in seven (9%) patients, diarrhea in nine (12%), liver toxicity in three (4%), nausea in three (4%), and appetite loss, fatigue, worsening performance status, thrombocytosis, proteinuria, pulmonary hypertension, and edema in one (1%) each. The incidences of discontinuation, interruption, and dose reduction and the therapeutic period showed no significant differences between patients with and without dyspnea. The present study showed no difference in the tolerability of nintedanib therapy according to the presence of dyspnea in patients with FILD.

## 4. Discussion

This study demonstrated that a substantial proportion of asymptomatic patients with FILD who require nintedanib therapy have significant exercise-induced oxygen desaturation. The outcomes after nintedanib therapy were better in patients without than with dyspnea, as evidenced by a lower incidence of progression, a slower decline in pulmonary function, and better survival of FILD even after adjustment for covariates.

Among the 18 patients with FILD without dyspnea, 4 (24%) showed a decrease in SpO_2_ to <90%, and 12 (71%) showed a decrease in SpO_2_ of >4% during the 6MWT. These cut-off levels of desaturation were associated with survival in patients with FILD [17,18]. Similarly, 6 (40%) of 18 patients who had FILD without dyspnea showed a CAT score of >10 as a prognostic factor for COPD [28]. The CAT score was reported as a predictor of OS in patients receiving oxygen therapy (i.e., patients with non-early-stage disease) in a previous report [27], but this finding has not been revealed in patients without dyspnea. The present study showed that asymptomatic patients with FILD have potential exercise-induced oxygen desaturation and high CAT scores. Moreover, in the comparative analyses adjusted for covariates as prognostic factors of FILD, patients without dyspnea had better outcomes after nintedanib therapy than those with dyspnea. These findings demonstrate that antifibrotic drugs can benefit asymptomatic patients with FILD and should not be avoided merely because of the absence of dyspnea. The mechanisms underlying the differences in outcomes after nintedanib therapy between patients with and without dyspnea are unknown. In the subgroup analysis of the SENSCIS phase 3 trial, there was no difference in the decrease over 1 year between the non-dyspnea and dyspnea placebo groups with SSc-ILD. These findings suggest that nintedanib can slow the decline in pulmonary function in patients who have FILD without dyspnea [15]. By contrast, the non-dyspnea group had a numerically smaller extent of fibrotic area and lower pulmonary function at baseline in this subgroup study [15]. The results of the present study, which show that patients without dyspnea have better outcomes after nintedanib therapy than those with dyspnea, might be associated with the possibility that patients with dyspnea have fewer established fibrotic lesions resistant to drug therapy. Moreover, initiating antifibrotic drugs for all asymptomatic patients with FILD may lead to excessive side effects and an economic burden for patients. Therefore, further research is needed to identify biomarkers to select asymptomatic patients with FILD who are suitable for early intervention with antifibrotic drugs. These issues should be addressed in future studies.

Although blood biomarkers, including C-reactive protein, KL-6, lactate dehydrogenase (LDH), and the monocyte count, were not associated with annual progression of FILD in the present study, they have been reported as biomarkers for predicting mortality, progression, and the development of AE [31,32,33,34,35]. Huang et al. [31] reported that high baseline plasma KL-6 levels are a risk factor for death, AE, and liver damage in IPF patients treated with nintedanib. Additionally, in our retrospective study, the effectiveness of nintedanib in suppressing a decline in D_LCO_ in patients with IPF was higher in patients with higher than lower serum KL-6 or LDH levels [32]. Further studies are needed to clarify serum biomarkers that predict the effectiveness of antifibrotic drugs in patients with FILD.

Although the diagnosis of IPF and a definite UIP pattern on chest computed tomography have been reported as prognostic factors of FILD [1,2,3,4,36], neither was associated with annual progression of FILD in our study. This result may be associated with the possibility that patients with progression were selected for nintedanib therapy. A previous study showed that OS was not significantly different between patients with progressive pulmonary fibrosis and those with IPF [6]. Another study showed that among patients with IPF, there was no significant difference in OS between those showing a definite UIP pattern and those showing a probable UIP pattern [36].

In the present study, there was no difference in nintedanib tolerability between patients with and without dyspnea, as demonstrated by comparable discontinuation rates and therapeutic period. This suggests that the presence of dyspnea may not be associated with the tolerability of nintedanib. Previous retrospective studies in Japan have identified higher age, lower body mass index, and worse performance status as risk factors for lower tolerability and safety of nintedanib, which could be mitigated by dose reduction from 300 to 200 mg/day [37,38,39]. The results of our study may have been influenced by the fact that there were no significant differences in age, body mass index, or percentage of patients with a reduced starting dose of nintedanib, because nintedanib therapy for patients with FILD tends to be started at an early disease stage in our hospital. Further studies involving more patients with severe disease may be needed to clarify the difference in tolerability between patients with and without dyspnea.

The present study had several limitations. First, the sample size was relatively small, and larger population studies are needed to validate these findings. Second, this was a retrospective, single-arm study. Although a randomized controlled trial would be the most appropriate design for evaluating the effectiveness and tolerability of nintedanib, conducting such a trial would be challenging. Nevertheless, the present study provides a rationale for planning future randomized controlled trials.

## 5. Conclusions

We showed that patients who had FILD without dyspnea have severe exercise-induced oxygen desaturation and better outcomes after nintedanib therapy than those with dyspnea, but there was no difference in tolerability of this therapy between the two groups. Treatment with antifibrotic drugs should not be avoided solely because of an absence of symptoms.

## Figures and Tables

**Table 1:** Patients’ characteristics.

	Overall	Non-Dyspnea Group	Dyspnea Group	*p* Value
Number	76	18 (24%)	58 (74%)	
Age (years)	72.2 (65.9–76.2)	70.8 (65.2–74.4)	72.5 (66.2–76.6)	0.17
Male gender	56 (74%)	17 (94%)	39 (69%)	0.030 *
FILD subtype (FILD/UC-ILD/ HP/CTD/SR-ILD)	53 (70%)/11 (15%)/ 7 (9%)/4 (5%)/1 (1%)	14 (77%)/1(6%)/ 2 (11%)/1(6%)/0	39 (67%)/10 (17%)/ 5 (9%)/3(5%)/1 (2%)	
Smoker	60 (79%)	17 (94%)	43 (74%)	0.097
Body mass index (kg/m^2^)	24.3 (22.0–26.7)	24.5 (22.0–26.3)	24.0 (21.8–27.0)	0.67
mMRC scale	1 (1–2)	0 (0–0)	1 (1–2)	<0.00010 *
GAP score (0 or 1/2 or 3/4 or 5/6 to 8)	9 (12%)/38 (50%) /28 (37%)/1 (1%)	3 (17%)/13 (72%) /2 (11%)/0	6 (10%)/25 (43%) /26 (45%)/1 (2%)	0.030 *
HRCT pattern				
Definite UIP	25 (33%)	7 (39%)	18 (31%)	0.57
Probable UIP	17 (22%)	5 (28%)	12 (21%)	
Indeterminate for UIP	21 (28%)	3 (17%)	18 (31%)	
Alternative diagnosis	13 (17%)	3 (17%)	10 (17%)	
Pulmonary function				
FVC (%)	81.5 (66.3–92.8)	84.2 (79.1–99.4)	78.8 (64.6–91.3)	0.025 *
D_LCO_ (%)	61.3 (48.4–72.6)	72.2 (63.4–84.8)	58.2 (43.5–67.8)	0.00070 *
Six-minutes walk test				
Lowest SpO_2_ (%)	90.0 (85.0–92.5)	92.0 (89.5–94.0)	88.0 (84.3–92.0)	0.0015 *
Less than 90%	35 (48%)	4 (24%)	31 (55%)	0.028 *
Decrease in SpO_2_ (%)	6.0 (4.0–10.5)	5.0 (2.5–6.5)	7.0 (5.0–11.5)	0.0068 *
More than 4%	62 (82%)	12 (71%)	50 (89%)	0.11
Walk distance (m)	375.0 (307.5–463.0)	420.0 (399.5–508.5)	359.0 (293.0–435.0)	0.0068 *
Serum biomarker				
CRP (mg/dL)	0.14 (0.070–0.43)	0.11 (0.050–0.16)	0.25 (0.073–0.48)	0.030 *
KL-6 (IU/mL)	782.0 (565.0–1233.0)	584.5 (437.3–869.0)	875.0 (601.5–1310.5)	0.014 *
LDH (IU/L)	210.0 (183.0–243.3)	200.0 (172.0–227.5)	218.5 (185.0–252.8)	0.13
Monocyte count (/uL)	414.5 (330.7–530.2)	370.9 (325.3–414.8)	457.3 (329.8–556.2)	0.054
CAT	14.0 (8.0–23.0)	6.0 (3.0–13.0)	17.0 (11.0–25.0)	0.00020 *
More than 10	48 (63%)	6 (40%)	42 (82%)	0.0025 *
Starting dose of nintedanib (mg/day) 200/300	7 (9%)/69 (91%)	1 (6%)/17 (94%)	6 (10%)/52 (90%)	1

Data are expressed as medians (25th to 75th percentiles of interquartile range) unless otherwise stated. FILD, fibrosing interstitial lung disease; IPF, idiopathic pulmonary fibrosis; UC-ILD, unclassifiable interstitial lung disease; HP, hypersensitivity pneumonia; CTD, connective tissue disease; SR-ILD, smoking-related interstitial pneumonia; mMRC, modified Medical Research Council; GAP, gender–age–physiology; UIP, usual interstitial pneumonia; CT, computed tomography; FVC, forced vital capacity; D_LCO_, diffusing capacity of the lung for carbon monoxide; 6MWT, 6 min walk test; CRP, C-reactive protein; KL-6, Krebs von den Lungen-6; LDH, lactate dehydrogenase; CAT, chronic obstructive pulmonary disease assessment test. * *p* < 0.05 is regarded as statistically significant.

**Table 2:** Patients’ outcomes.

	Overall	Non-Dyspnea Group	Dyspnea Group	*p* Value
Number	76	18 (24%)	58 (74%)	
Overall survival (days)	861.0 (671.5–1488.5)	1382.5 (822.8–1540.3)	780.0 (604.0–1341.5)	0.017 *
Mortality from any cause	24 (32%)	2 (11%)	22 (38%)	0.042 *
Mortality due to FILD	17 (22%)	0	17 (29%)	0.0080 *
Annual disease progression	34 (45%)	3 (17%)	31 (53%)	0.0070 *
More than 10% decrease in FVC	12 (16%)	2 (11%)	10 (17%)	0.72
More than 15% decrease in D_LCO_	30 (41%)	3 (17%)	27 (48%)	0.026 *
Developing acute exacerbation	2 (3%)	0	2 (3%)	1
ILD-specific mortality	2 (3%)	0	2 (3%)	1
Immunosuppressive therapy over 180 days	12 (16%)	1 (6%)	11 (19%)	0.27

Data are expressed as median (25th to 75th percentiles of interquartile range) unless otherwise stated. FILD, fibrosing interstitial lung disease; FVC, forced vital capacity; D_LCO_, diffusing capacity of the lung for carbon monoxide; AE, acute exacerbation; ILD, interstitial lung disease. * *p* < 0.05 is regarded as statistically significant.

**Table 3:** (**A**) Univariate analysis of factors associated with annual progression of FILD. (**B**) Multivariate analysis of factors associated with annual progression of FILD with backward elimination. (**C**) Multivariate analysis of factors associated with annual progression of FILD without backward elimination.

	Change in Variables	OR	95% CI	S.E.	*p* Value
(**A**)					
Age (years)	5.0	1.07	0.79–1.5	0.0308	0.68
Male gender		0.43	0.15–1.2	0.266	0.11
ILD subtype					
IPF		1.8	0.36–9.4	0.258	0.25
Fibrotic HP		1.7	0.36–9.4	0.401	0.49
UC-ILD		0.41	0.084–1.6	0.361	0.22
CT-definite UIP pattern		1.5	0.59–4.1	0.246	0.37
Smoker		0.56	0.18–1.7	0.284	0.30
Body mass index (kg/m^2^)	10.0	0.84	0.25–2.8	0.0616	0.78
mMRC scale (0/1/2/3/4)					
0 (without dyspnea)		0.17	0.037–0.60	0.343	0.011 *
<2		0.37	0.12–1.1	0.027	0.067
<3		0.36	0.071–1.5	0.375	0.17
GAP score	1.0	1.1	0.82–1.6	0.175	0.43
Pulmonary function					
FVC (%)	10.0	0.82	0.62–1.1	0.0137	0.16
D_LCO_ (%)	10.0	0.88	0.68–1.1	0.0127	0.32
Six-minutes walk test					
Lowest SpO_2_ (%)	1.0	0.90	0.82–0.98	0.0451	0.021 *
Decrease in SpO_2_ (%)	1.0	1.1	1.0–1.3	0.0528	0.029 *
Walk distance (m)	10.0	0.95	0.90–0.99	0.00241	0.032 *
Serum biomarker					
CRP (mg/dL)	1.0	1.1	0.42–3.0	0.458	0.82
KL-6 (IU/mL)	100.0	1.008	0.95–1.1	0.000265	0.75
LDH (IU/L)	10.0	1.1	1.0–1.2	0.00508	0.055
Monocyte count (/uL)	10.0	1.02	0.99–1.05	0.00137	0.22
CAT	5.0	1.3	0.99–1.7	0.0268	0.065
Immunosuppressant use		0.57	0.14–2.0	0.331	0.39
(**B**)					
mMRC scale 0 (without dyspnea)		0.17	0.045–0.67	0.343	0.011 *
Age (years)	5.0				N.S.
Male gender					N.S.
IPF					N.S.
6 min walk distance (m)	10.0				N.S.
Lowest SpO_2_ (%)	1.0				N.S.
Decrease in SpO_2_ (%)	1.0				N.S.
FVC (%)	10.0				N.S.
D_LCO_ (%)	10.0				N.S.
(**C**)					
mMRC scale 0 (without dyspnea)		0.14	0.022–0.83	0.463	0.031 *
Age (years)	5.0	0.90	0.61–1.3	0.0379	0.58
Male gender		0.64	0.15–2.7	0.367	0.54
IPF		4.4	1.1–16.6	0.341	0.031 *
6 min walk distance (m)	10.0	0.98	0.91–1.04	0.00330	0.45
Lowest SpO_2_ (%)	1.0	0.99	0.73–1.3	0.151	0.98
Decrease in SpO_2_ (%)	1.0	1.01	0.97–1.1	0.168	0.60
FVC (%)	10.0	0.81	0.56–1.1	0.0178	0.24
D_LCO_ (%)	10.0	1.1	0.75–1.7	0.0203	0.56

(**A**) OR, odds ratio; SE, standard error; confidence interval; FILD, fibrosing interstitial lung disease; ILD, interstitial lung disease; IPF, idiopathic pulmonary fibrosis; UC-ILD, unclassifiable interstitial lung disease; HP, hypersensitivity pneumonia; CTD, connective tissue disease; mMRC, modified Medical Research Council; GAP, gender–age–physiology; UIP, usual interstitial pneumonia; CT, computed tomography; FVC, forced vital capacity; D_LCO_, diffusing capacity of the lung for carbon monoxide; 6MWT, 6 min walk test; SpO_2_, saturation of percutaneous oxygen; CRP, C-reactive protein; KL-6, Krebs von den Lungen-6; LDH, lactate dehydrogenase; CAT, chronic obstructive pulmonary disease assessment test. ORs are the values when explanatory variables fluctuate within the range of change. * *p* < 0.05 is regarded as statistically significant. (**B**) OR, odds ratio; SE, standard error; confidence interval; FILD, fibrosing interstitial lung disease; mMRC, modified Medical Research Council; IPF, idiopathic pulmonary fibrosis; SpO_2_, saturation of percutaneous oxygen; FVC, forced vital capacity; D_LCO_, diffusing capacity of the lung for carbon monoxide; ORs are the values when explanatory variables fluctuate within the range of change. * *p* < 0.05 is regarded as statistically significant. (**C**) OR, odds ratio; SE, standard error; confidence interval; FILD, fibrosing interstitial lung disease; mMRC, modified Medical Research Council; IPF, idiopathic pulmonary fibrosis; SpO_2_, saturation of percutaneous oxygen; FVC, forced vital capacity; D_LCO_, diffusing capacity of the lung for carbon monoxide; ORs are the values when explanatory variables fluctuate within the range of change. * *p* < 0.05 is regarded as statistically significant.

**Table 4:** Comparison of tolerability for nintedanib therapy with or without dyspnea.

	Overall	Non-Dyspnea Group	Dyspnea Group	*p* Value
Number	76	18 (24%)	58 (74%)	
Discontinuation				
Overall period/all cause	26 (34%)	6 (33%)	20 (34%)	0.78
Overall period/due to side effect	20 (26%)	4 (22%)	16 (28%)	0.77
For 1 year/all cause	10 (13%)	1 (6%)	9 (16%)	0.44
For 1 year/due to side effect	10 (13%)	1 (6%)	9 (16%)	0.44
Interruption	27 (36%)	8 (44%)	19 (33%)	0.41
Dose reduction	52 (68%)	15 (83%)	37 (64%)	0.15
Cause of discontinuation				
Worsening ILD	7 (9%)	2 (11%)	5 (9%)	
Diarrhea	9 (12%)	2 (11%)	7 (12%)	
Liver toxicity	3 (4%)	1 (6%)	2 (4%)	
Nausea	3 (4%)	1 (6%)	2 (4%)	
Appetite loss	1 (1%)	0	1 (2%)	
Fatigue	1 (1%)	0	1 (2%)	
Worsening of PS	1 (1%)	0	1 (2%)	
Thrombocytopenia	1 (1%)	0	1 (2%)	
Proteinuria	1 (1%)	0	1 (2%)	
Developing PH	1 (1%)	0	1 (2%)	
Edema	1 (1%)	0	1 (2%)	

OR, odds ratio; SE, standard error; CI, confidence interval; IPF, idiopathic pulmonary fibrosis; mMRC, modified Medical Research Council; SpO_2_, saturation of percutaneous oxygen; FVC, forced vital capacity; D_LCO_, diffusing capacity of the lung for carbon monoxide; CAT, chronic obstructive pulmonary disease assessment test.

## Data Availability

The datasets used and/or analyzed during the current study are available from the corresponding author on reasonable request.
